# Supplementary material for: Protein polarization driven by nucleoid exclusion of DnaK(HSP70)–substrate complexes
Source: Nat Commun. 2018 May 23;9:2027. doi: 10.1038/s41467-018-04414-2 (PMC5966378; doi:10.1038/s41467-018-04414-2)
Supplement: Supplementary file 1 — Supplementary Information [file 41467_2018_4414_MOESM1_ESM.pdf]

Supplementary Information

Supplementary Methods
Supplementary Figures 1-11
Supplementary References

Protein polarization driven by nucleoid exclusion of DnaK(HSP70)-substrate complexes

Collet C et al. 2018

Supplementary Methods

Preparation of Colicin E2 and bacterial treatment

Colicin E2 was prepared as previously described[1]. Briefly, the colicinogenic strain A798 was grown to $OD_{600\ nm}$ = 0.1 in LB medium at 37°C in a shaking incubator. Following addition of mitomycin at a final concentration of 2 µg/ml, the bacterial culture was incubated until a final $OD_{600\ nm}$ = 0.5. Bacteria were pelleted by centrifugation for 15 min at 8000 xg at 4°C, and the Colicin E2-containing supernatant was transferred to a fresh tube. Chloroform was added at a final concentration of 0.01%, and the supernatant (E2 SN) was stored at 4°C. As expected, the E2 SN showed bactericidal activity when used at dilutions as low as 10^{-4} in LB. Bacterial DNA degradation was observed after 15 min when E2 SN was used at a dilution of 1:2, and this is the dilution used for E2 SN treatments. Following one-hour induction of Ci_v or DnaK-RFP with 100 nM IPTG, Colicin E2 and ciprofloxacin (25 µg/ml) were added to exponentially grown bacteria.

Image analysis

The size and intensity of Ci polar area were quantified from fluorescent images using the Metamorph 7.7 software. Briefly, average intensity projection of 200 nm-spaced planes from Z-stacks of images acquired using spinning disk confocal fluorescence microscopy were subjected to automatic thresholding of light objects using the software implemented function. Because of the sharp contrast of Ci fluorescence, this method enabled the unambiguous detection and determination of the size of the Ci-containing regions. The average fluorescence intensity of the Ci polar region was corrected for background by subtracting the average fluorescence intensity of a non-relevant area devoid of bacteria. The values were normalized by dividing by the average intensity at the Ci polar region determined for untreated bacteria. The results, expressed as an average value per bacteria, were representative of 2 independent experiments. Untreated: 80 bacteria; + ciprofloxacin: 177; + Colicin E2: 40 bacteria.

Supplementary Figures

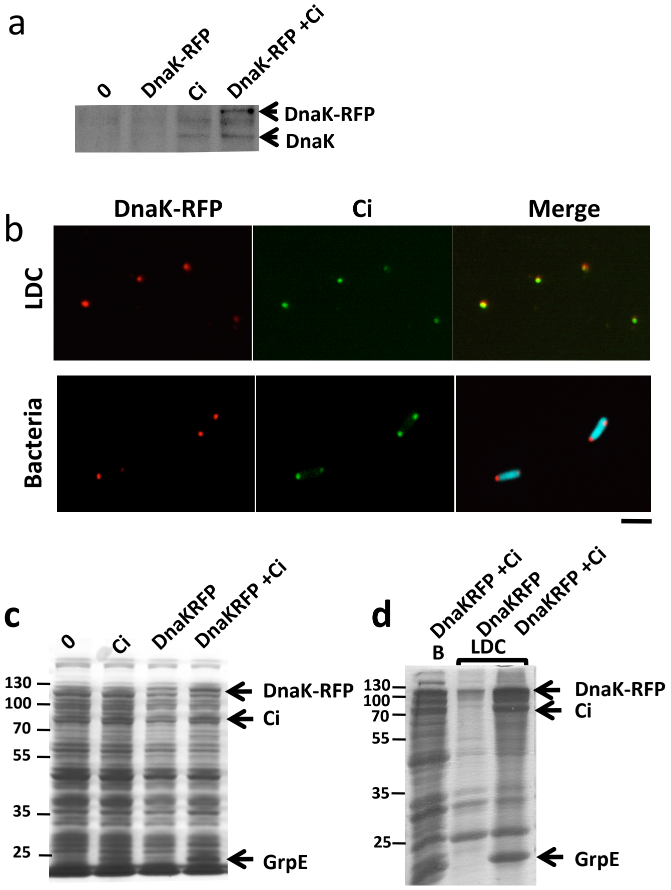

Supplementary Figure 1. DnaK-bound to Ci forms large polar complexes.

(a) DnaK-RFP binds to Ci. Bacterial lysates were subjected to cross-linking and anti-IpaC immunoprecipitation. Immunoprecipitates were analyzed by anti-DnaK Western blotting. MC4100 (0), or MC4100 expressing the indicated constructs. (**b**) Cross-linked LDCs were prepared from MC4100/pSUCi pDnaK-RFP, using a procedure for the purification of inclusion bodies (Materials and Methods). Samples were analyzed by fluorescence microscopy. LDCs are visualized as large particles with size reaching 700 nm, containing Ci and DnaK-RFP. Such particles correspond to the polar fractions observed in the parental strain. Scale bar = 2 µm. (**c**, **d**) Coomassie blue analysis of 10 % (**c**) and 7.5 % polyacrylamide SDS-PAGE gels of total lysates (**c** and **d**, lane B) and purified LDCs (Materials and Methods).

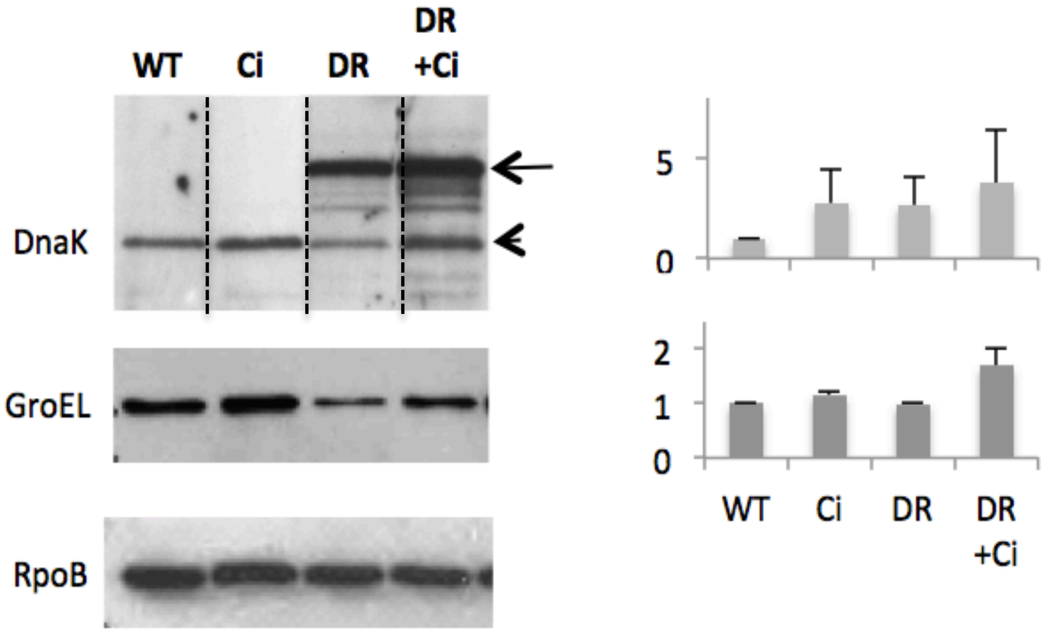

Supplementary Figure 2. Ci induces a heat-shock like response

Samples of bacteria grown at 30°C to exponential phase and further incubated for 1 hour in the presence of 100 μM IPTG to induce the expression of the indicated recombinant proteins were analyzed by Western blot with DnaK, GroEL and RpoB antibodies (Left panels). Arrow: DnaK-RFP; arrowhead: DnaK. Right panels: histograms showing the quantification of the integrated band intensities ± SEM from three independent experiments. WT: MC4100; Ci: MC4100/pSUCi; DR: MC4100/pDnaK-RFP; DR+Ci:MC4100/pSUCi pDnaK-RFP.

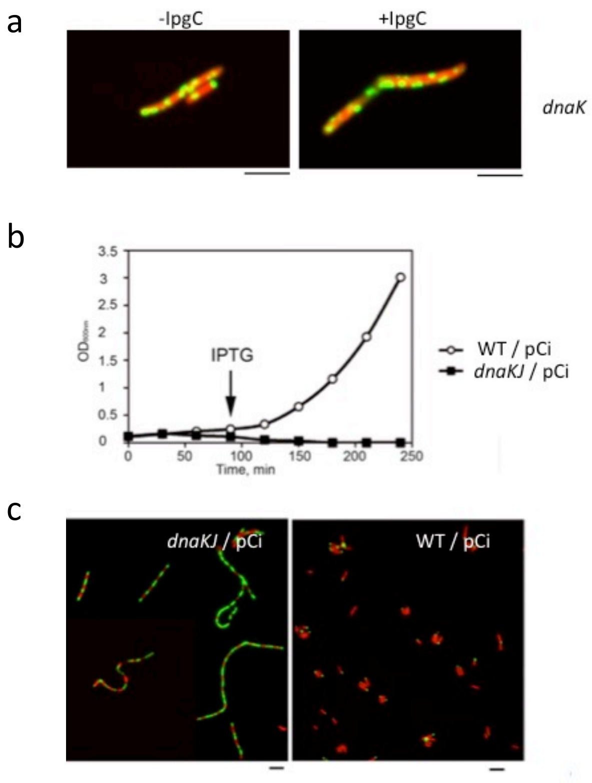

Supplementary Figure 3. Ci expression is lethal in a *dnaKJ* mutant.

Bacteria were grown at 30°C until early exponential phase and Ci expression was induced by the addition of 2 mM arabinose (**a**) or 100 µM IPTG (**b**, **c**). Expression of the IpgC chaperone was induced with 500 µM IPTG (**a**). (**a**, **c**) Representative fluorescence micrographs of the indicated bacteria 60 min following Ci induction. Red: DAPI staining of nucleoids; green: Ci fluorescence. Scale bar = 2 µm. (**a**) MC4100/pSUCi/P17A (+IpgC) or MC4100/pSUCi (-IpgC). Expression of the IpgC chaperone does not prevent Ci aggregation. (**b**) Bacterial growth was monitored by following the optical density at 600 nm. The arrow indicates the addition of IPTG. Open circles: WT/pCi; solid squares: *dnaK*/pCi. (**c**) Note the formation of filamentous bacteria containing mutually excluding area of Ci and DNA in *dnaK*/pCi associated with bacterial growth arrest.

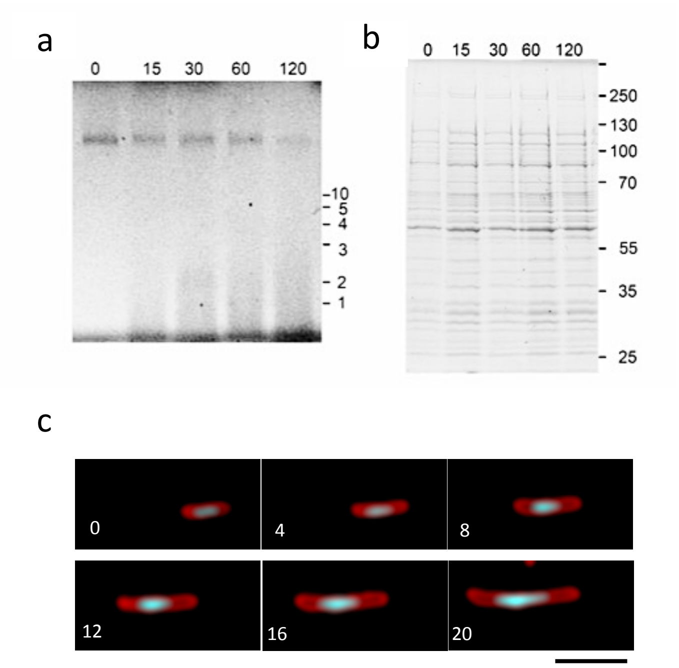

Supplementary Figure 4. Time-lapse analysis of Colicin E2 effects on the bacterial nucleoid.

Bacteria were grown to exponential phase, Colicin E2 was added, and samples were further incubated for the time indicated in min. Bacteria were lysed and: (**a**) total DNA was analyzed on a 0.8 % agarose gel and ethidium bromide staining. The migration of molecular weight standards in kilobases is indicated; (**b**) proteins were analyzed by SDS-PAGE on a gel containing 10 % polyacrylamide followed by Coomassie blue staining. The migration of molecular weight standards in kDa is indicated. DNA degradation is detectable after 15 min incubation. (**c**) Representative time serial micrographs of wild-type *E. coli* grown on an agarose pad at 37°C in the presence of DAPI to stain the nucleoid. The time following addition of Colicin E2 is indicated in min above the panels. Red: phase contrast acquisition processed with the ImageJ "Find edges" plug-in; blue: DAPI fluorescence. Scale bar = 5 µm. Data shown are representative of at least 3 independent experiments.

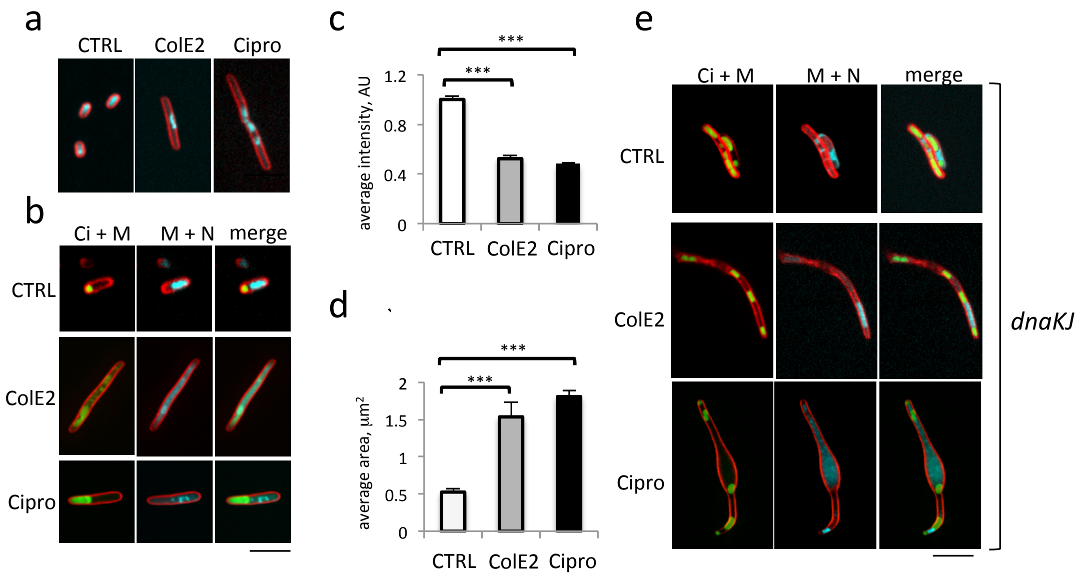

Supplementary Figure 5. LDCs are confined to the pole by nucleoid occlusion. Bacterial strains were treated with Colicin E2 (ColE2) or ciprofloxacin at 25 µg/ml final concentration (Cipro), or in medium alone (CTRL) for 2 hours. Samples were fixed and analyzed by fluorescence microscopy. (**a, b, e**) Representative micrographs of: (**a**) MC4100; (**b**) MC4100/pCi; (**e**) isogenic *dnaKJ*/pCi. Green: Ci; red: FM4-64-membrane staining (M); cyan: DAPI staining (N). Scale bar = 2 µm. The average fluorescence intensity (**c**) and average area (**d**) ± SEM of the Ci region were quantified for at least 30 bacteria from 3 independent experiments. Dunn test. ***: $p < 10^{-3}$. Statistical difference relative to untreated bacteria.

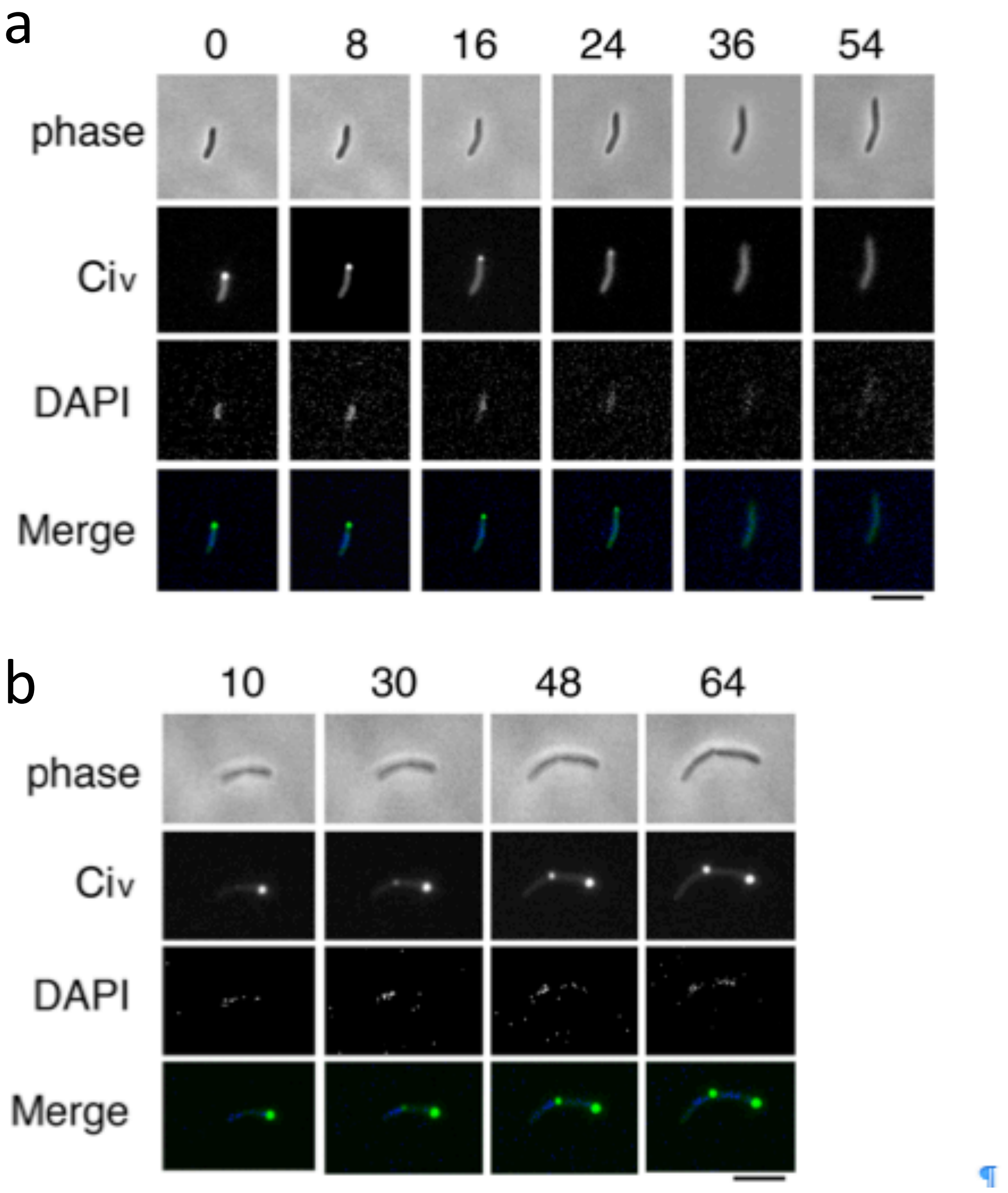

Supplementary Figure 6. Time-lapse analysis of ciprofloxacin effects on Ci_v localization. Bacteria were grown to exponential phase and Ci_v expression was induced for 60 min with IPTG prior to plating onto agarose pad. Live fluorescence microscopy analysis in the presence (**a**) or absence (**b**) of ciprofloxacin. Representative time serial micrographs of WT/pCi_v grown in the presence of DAPI to stain the nucleoid. The time following addition of ciprofloxacin is indicated in min above the panels. Phase: phase contrast acquisition. Scale bar = 5 μm. Note that while in the absence of antibiotic, Ci_v remains polar during bacterial growth, ciprofloxacin induces a diffuse localization of Ci_v associated with the disappearance of the nucleoid. Data shown are representative of at least 3 independent experiments.

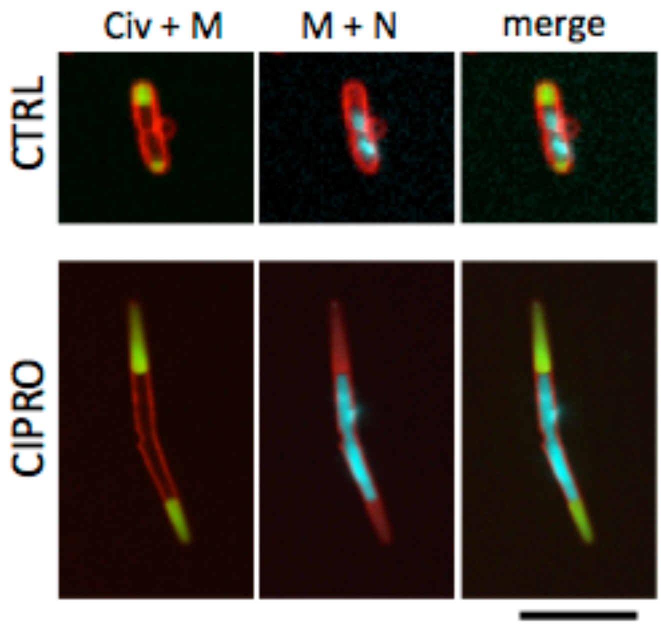

Supplementary Figure 7. Ciprofloxacin treatment leads to the formation of fusiform bacteria.

Bacteria were grown to exponential phase and Ci_v expression was induced for 60 min with IPTG, Bacteria were further incubated in the absence (CTRL) or presence of ciprofloxacin (Cipro). Samples were fixed and processed for fluorescence microscopy. Staining: M: membrane (red); N: DAPI staining (cyan); Ci (green). Ciprofloxacin-treated cells form extended and fusiform poles filled with Ci. Scale bar = 5 μm. Data shown are representative of at least 3 independent experiments.

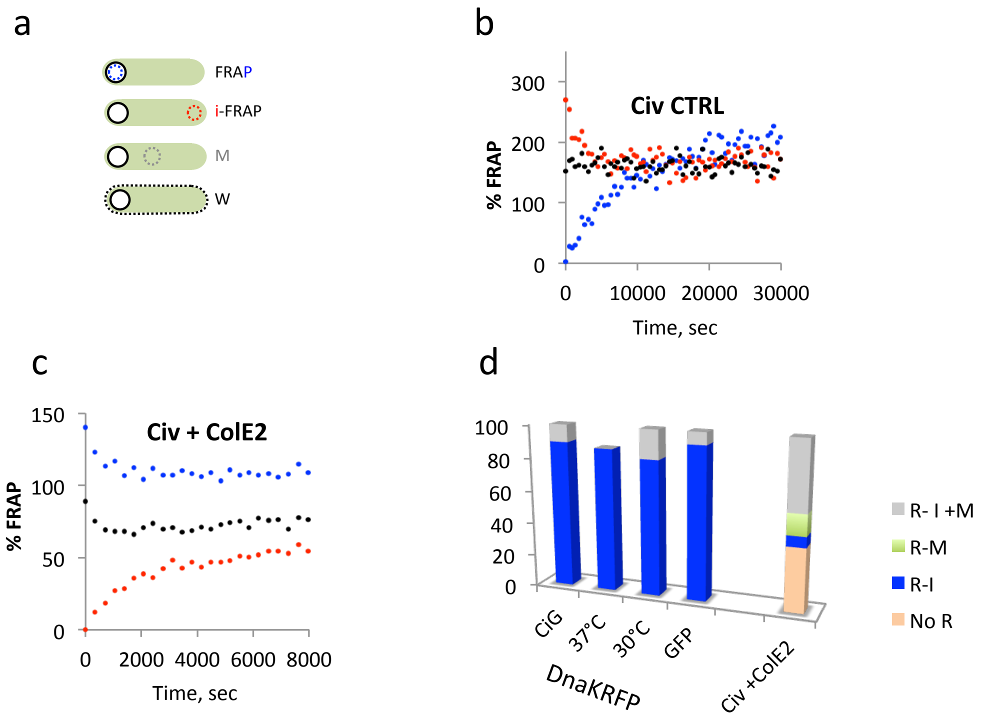

Supplementary Figure 8. Colicin E2 treatment leads to recovery from the mid-body region.

(**a**) Scheme of the analytical procedure. Empty circle: photobleached area. Dotted area: region subjected to FRAP analysis. (**b**, **c**) Representative fluorescence recovery kinetics of FRAP for control MC4100/pCi$_v$ (Civ CTRL), or treated with Colicin E2 (Civ+ColE2). (**d**) Recovery kinetics were analyzed as a function of the region showing a mirror decrease in fluorescence. Recovery from: R-I: the opposite pole; R-M: the mid body; R-I + M; the opposite pole and the mid body. No-R: no recovery. For each strain, the percent of recovery events was determined from at least 30 bacteria in 3 independent experiments. Wild-type bacteria expressing Ci, DnaK-RFP grown at 30°C or 37°C, Ci$_v$ or Ci$_v$ treated with Colicin E2. In control cells, for Ci or DnaK-RFP, FRAP occurs predominantly from the opposite pole as illustrated in b. In Colicin E2-treated cells, however, ca 40 % of bacterial cells do not show recovery and 55 % show recovery from the mid-body or from the mid-body and the opposite pole, consistent with LDCs diffusing in the mid-body region following DNA degradation.

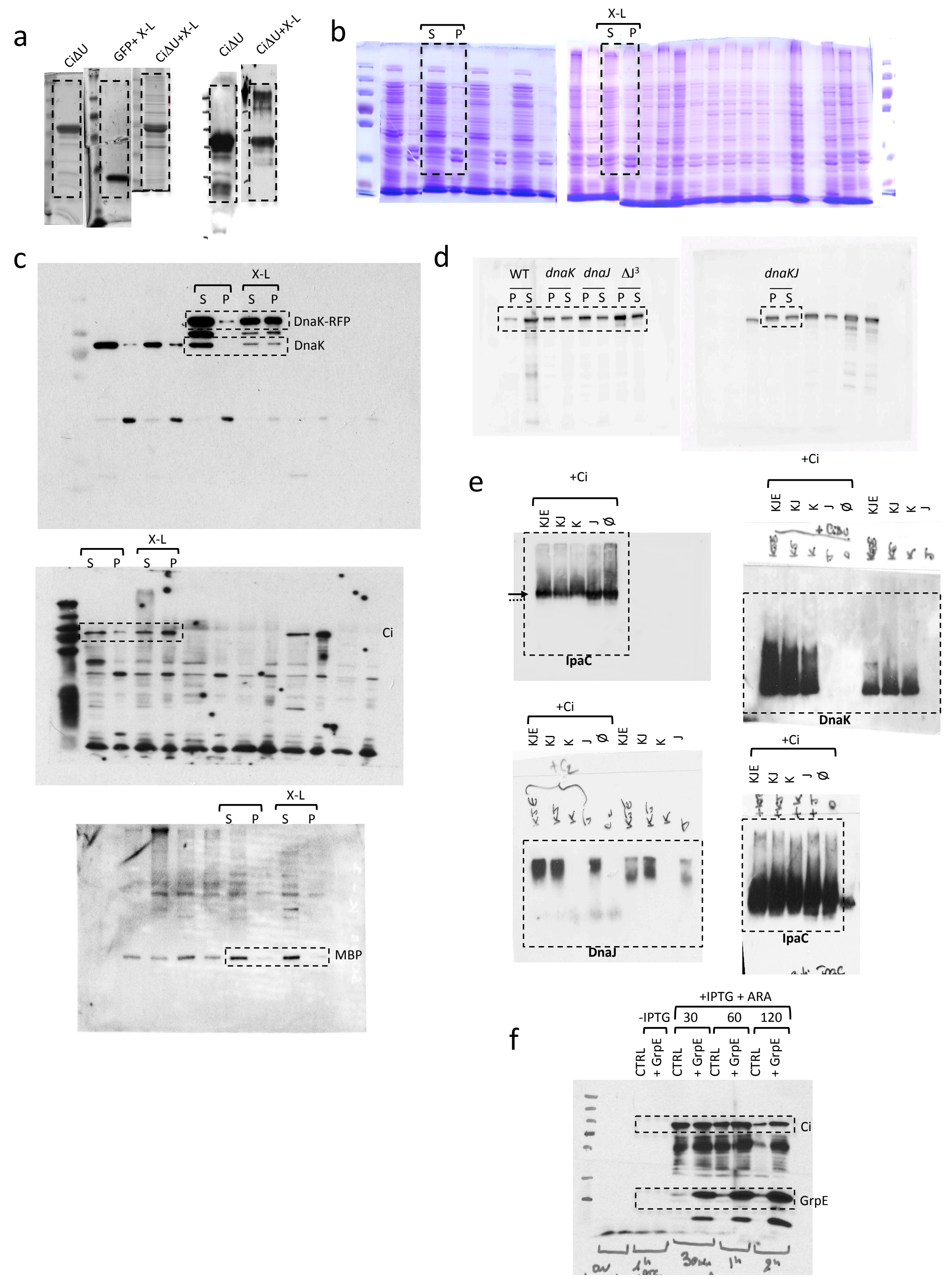

Supplementary Figure 9. Uncropped coomassie gels and Western blots showing molecular weight markers. (**a**) Uncropped Western blots showing molecular weight markers from Figure 1c. (**b**) Uncropped coomassie gels showing molecular weight markers from Figure 1d. (**c**) Uncropped Western blots showing molecular weight markers from Figure 1e. (**d**) Uncropped Western blots corresponding to Figure 3c. (**e**) Uncropped Western blots corresponding to Figure 7a. (**f**) Uncropped Western blot corresponding to Figure 8a.

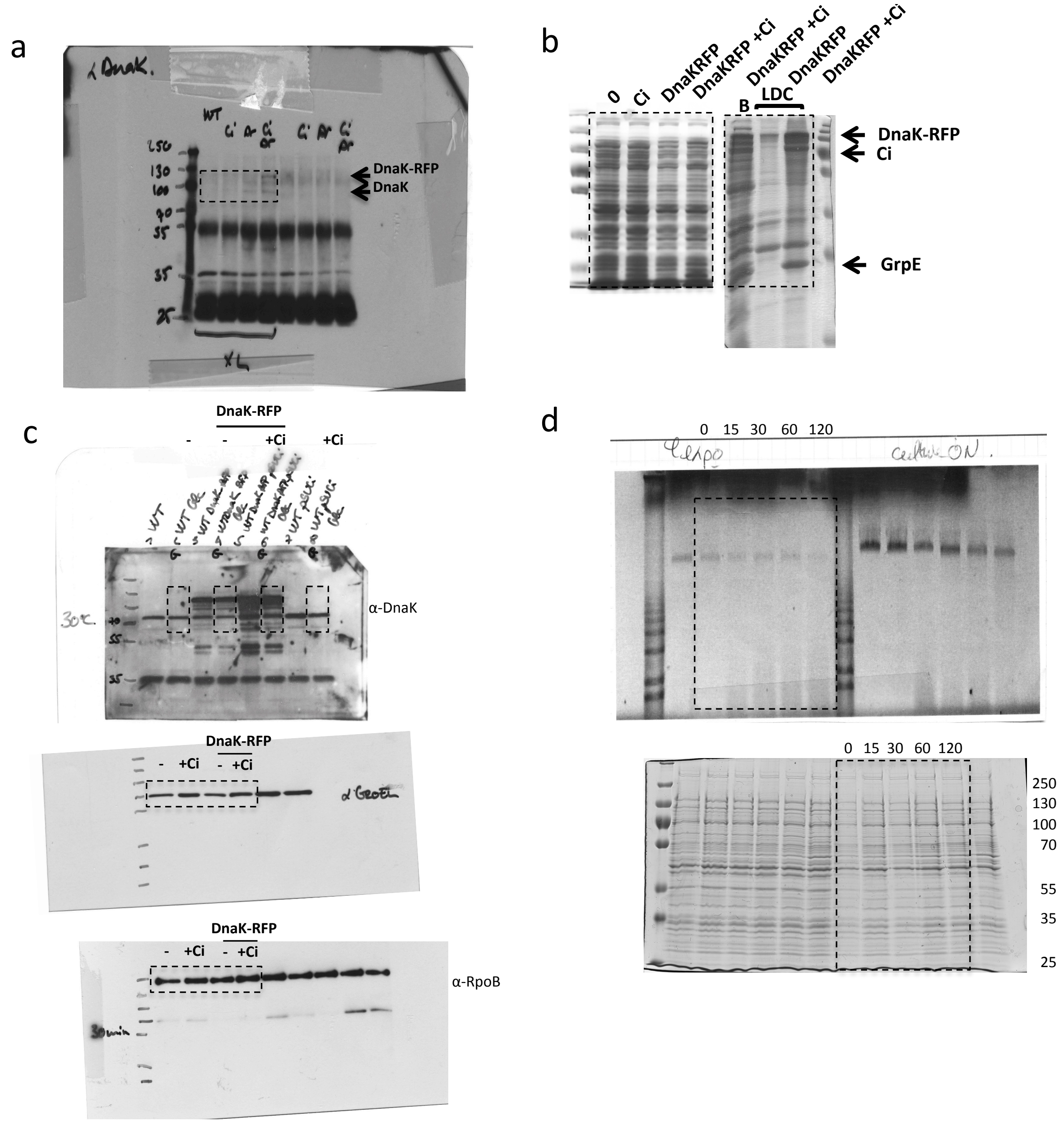

Supplementary Figure 10. Uncropped agarose gels, coomassie gels and Western blots showing molecular weight markers. (**a**) Uncropped Western blot showing molecular weight marker from Supplementary Figure 1a. (**b**) Uncropped coomassie gels showing molecular weight markers from Supplementary Figure 1c and d. (**c**) Uncropped Western blots corresponding to Supplementary Figure 2. The lane order of the DnaK blot is rearranged in Supplementary Figure 2. (**d**) Uncropped agarose gel and Coomassie gel showing molecular weight markers from Supplementary Figure 4a and b.

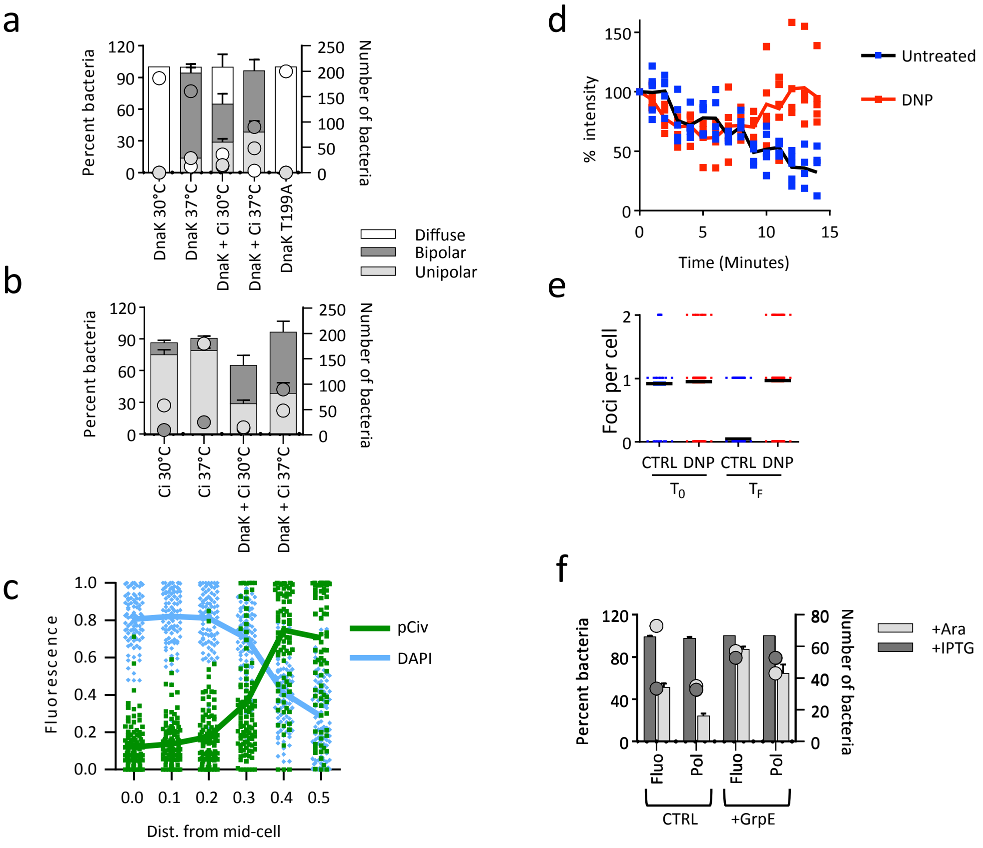

Supplementary Figure 11. Graphs from text showing data distribution. (**a, b**) Circles indicate the number of bacteria with diffuse, bipolar or unipolar localization of DnaK-RFP (**a**) or Ci (**b**) used to generate the stacked histogram in Figure 2b. (**c**) Circles indicate the number of bacteria with filled (black), unipolar (grey), or patches (dark grey) of Ci fluorescence used to generate the stacked histogram from Figure 3b. (**d**) Graph showing fluorescence intensity relative to the cell major axis, the squares represent individual measurements used to calculate the mean in Figure 4b and the line connects the mean fluorescence values. (**e**) LDC fluorescence intensity normalized to initial fluorescence (% intensity), the squares represent individual data measurements used to calculate the mean in Figure 4h and the line connects the mean values. (**f**) Number of foci in the presence (Red dots; DNP) or absence (Blue dots ; CTRL) of DNP at the onset of analysis (T_0) or following 60 min incubation (T_F) (foci were counted in 278, 352, 1096 and 486 bacteria from CTRL and DNP (T_0), CTRL and DNP (T_F) respectively; N = 3), used to calculate the mean ± SEM (black lines) shown in Figure 4i. (**g**) Circles indicate the number of bacteria (>30 bacteria, N=3) with fluorescent signal (Fluo), and bacteria with polar Ci signal (Pol), used to calculate mean ± SEM (bar graph) shown in Figure 8c.

Supplementary Reference

1. Herschman HR, Helinski DR. Purification and characterization of colicin E2 and colicin E3. *J Biol Chem* **242**, 5360-5368 (1967).
